# Risk of Herpes Zoster in Relation to Body Mass Index Among Residents Aged ≥50 Years: The Shozu Herpes Zoster Study

**DOI:** 10.2188/jea.JE20200473

**Published:** 2022-08-05

**Authors:** Kazuhiro Kawahira, Hironori Imano, Keiko Yamada, Yukiko Takao, Yasuko Mori, Hideo Asada, Yoshinobu Okuno, Koichi Yamanishi, Hiroyasu Iso

**Affiliations:** 1 Public Health, Department of Social Medicine, Osaka University Graduate School of Medicine, Osaka, Japan; 2 Cosmo Medical Clinic, Osaka, Japan; 3 Department of Psychology, McGill University, Québec, Canada; 4 Department of Anesthesiology and Pain Medicine, Juntendo University Faculty of Medicine, Tokyo, Japan; 5 Graduate School of Medicine, Center for Infectious Diseases, Kobe University, Hyogo, Japan; 6 Department of Dermatology, Nara Medical University School of Medicine, Nara, Japan; 7 Osaka Institute of Public Health, Osaka, Japan; 8 The Research Foundation for Microbial Diseases of Osaka University, Osaka, Japan; 9 Department of Public Health Medicine, Faculty of Medicine, University of Tsukuba, Ibaraki, Japan

**Keywords:** herpes zoster, body mass index, overweight, cohort study

## Abstract

**Background:**

The impact of body mass index on incidence of herpes zoster is unclear. This study investigated whether body mass index was associated with a history of herpes zoster and incidence during a 3-year follow-up, using data from a prospective cohort study in Japan.

**Methods:**

In total, 12,311 individuals were included in the cross-sectional analysis at baseline, of whom 1,818 with a history of herpes zoster were excluded from the incidence analysis, leaving 10,493 individuals. Body mass index (kg/m^2^) was classified into three categories (underweight: <18.5; normal: 18.5 to <25; and overweight: ≥25). To evaluate the risk of herpes zoster, we used a logistic regression model for prevalence and a Cox proportional hazard regression model for incidence.

**Results:**

Being overweight or underweight was not associated with herpes zoster prevalence at baseline. The multivariate hazard ratios of herpes zoster incidence for overweight versus normal-weight groups were 0.67 (95% confidence interval, 0.51–0.90) in all participants, and 0.57 (95% confidence interval, 0.39–0.83) in women, with no significant difference for men.

**Conclusion:**

Being overweight was associated with a lower incidence of herpes zoster than being normal weight in older Japanese women.

## INTRODUCTION

Varicella-zoster virus is the pathogen that causes herpes zoster (HZ) and typically causes varicella rash. Reactivation of latent varicella-zoster virus dominant in dorsal root ganglia occurs after its primary infection.^1^^,^^2^ The incidence and severity of HZ are known to develop because of the reduction of immunological function with age.^3^

The epidemiology of incidence and risk factors for HZ has been assessed by several studies in the United Kingdom,^4^ United States,^5^^,^^6^ Canada,^7^ and Japan.^8^ However, most of the studies were cross-sectional, except for one Japanese cohort study.^8^ Risk factors for HZ have been reported to be age, sex, ethnicity, genetic susceptibility, cell-mediated immune disorders, mechanical trauma, and psychological stress.^6^^,^^9^ Physicians and primary school teachers, who had repeatedly contact with varicella patients, had a lower risk of developing HZ, probably because their cell-mediated immunity was enhanced.^6^^,^^10^ Use of varicella vaccine to enhance varicella-zoster virus-specific cell-mediated immunity has been shown to prevent the development of HZ in older people.^11^^,^^12^ A previous study of 290 volunteers aged 40–45 years showed that body mass index (BMI) was positively associated with selected cell-mediated immunity parameters.^13^ We therefore hypothesized that BMI may have some impact on the development of HZ.

The Shozu Herpes Zoster (SHEZ) Study was a 3-year prospective cohort study that aimed to determine the incidence and risk factors for HZ among a general Japanese population.^14^ In this study, we used data from the SHEZ Study to investigate whether BMI was associated with HZ prevalence at baseline and incidence during follow-up.

## METHODS

### Study cohort

The SHEZ Study is a 3-year community-based prospective cohort study in Shozu County, Kagawa Prefecture. It aimed to examine the incidence and risk factors for HZ. The detailed methods of this study have been described previously.^14^ This study used data from the SHEZ Study, which was conducted in accordance with the Ethical Guidelines for Epidemiological Research and the Ethical Guideline for Clinical Studies after obtaining written informed consent from all participants. The SHEZ Study was approved by the Ethics Committee of the Research Foundation for Biomedical Diseases of Osaka University, Osaka University (ethical approval number 18384), the National Institute of Biomedical Innovation, and Nara Medical University.

### Study population

**Figure 1.**
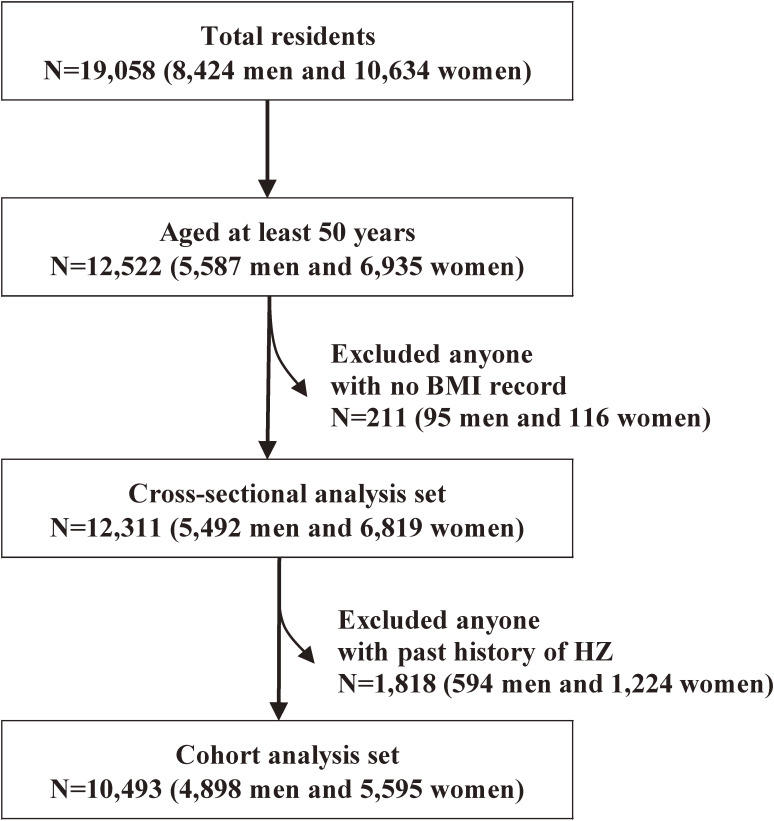
Flow chart for the study

The study participants were all residents of Shozu County (Shodoshima and Teshima Islands in Kagawa Prefecture) who were recruited to this study from December 2008, as described previously.^14^ HZ is commonly observed in older people,^1^^,^^15^ and therefore the study only included people aged ≥50 years. From a total of 19,058 (8,424 men and 10,634 women) residents, 12,522 (5,587 men and 6,935 women) aged ≥50 years were registered in the SHEZ Study. Of these, 211 individuals (95 men and 116 women) with no BMI data were excluded from this study. This left 12,311 individuals (5,492 men and 6,819 women) for inclusion in the cross-sectional analysis for prevalence of HZ at baseline. Of those 12,311 individuals, we further excluded 1,818 individuals (594 men and 1,224 women) with a history of HZ before the start of the study, leaving 10,493 individuals (4,898 men and 5,595 women) for inclusion in the analysis of incidence of HZ. The participants’ enrollment process is shown in a flow chart (Figure 1).

During the 3-year follow-up, 584 people (338 men and 246 women) died, 58 people (18 men and 40 women) were lost to follow-up, 43 persons (18 men and 25 women) withdrew, 39 people (12 men and 27 women) moved away, and one woman developed dementia.

### Baseline information obtained at enrollment

Participants were recruited between December 2008 and November 2009, and the baseline survey was conducted at registration. At the time of enrollment, each participant underwent an interview, and completed a self-administered health questionnaire including information about lifestyle habits (eg, smoking and drinking), and social/psychological factors. The interview was conducted by research physicians to collect information about past history of HZ and other diseases, and family history of HZ.^14^^,^^16^

BMI was calculated as the body weight divided by the square of the height (both self-reported), expressed in units of kg/m^2^. It was classified into three categories: underweight (<18.5 kg/m^2^), normal (18.5 to <25 kg/m^2^) or overweight (≥25 kg/m^2^).

### Surveillance for incidence of HZ

We conducted active surveillance for the incidence of HZ via monthly telephone surveys using an established script during the follow-up period.^14^ Participants were instructed to telephone the onsite study office if they showed any symptoms of herpes zoster, such as skin rash or pain, between the monthly telephone calls. The site office was open 24 hours a day. The study staff asked anyone with symptoms to visit a clinic or hospital for diagnosis and any treatment, and would accompany patients or meet them at the clinic or hospital. When a patient with suspected HZ visited a clinic or hospital without calling the study office, physicians at that clinic called the study office.

The research team evaluated pain, photographed skin areas with rash or pain, and collected venous blood to check for varicella-zoster virus using polymerase chain reaction (PCR) analysis at the first visit. A final diagnosis was made by the outcome evaluation team of three dermatologists using this clinical information.

### Statistical analysis

To examine differences in baseline characteristics of the participants by BMI category (underweight, normal weight, and overweight) in total participants and both sexes, we used Student’s *t*-test for continuous variables, such as age and BMI, and the chi-square test for categorical variables, using the normal-weight group as a reference.

In the cross-sectional analysis for the prevalence of HZ at baseline, we used logistic regression analysis to estimate the odds ratios (ORs) and 95% confidence intervals (CIs) and identify any association between BMI category and a past history of HZ after adjustment for age and sex. We subsequently adjusted other confounding variables including smoking (≥one cigarette per day or not), drinking (>23 g of alcohol per day or not), exercise habit (≥3 hours/week or not), walking (≥1 hour/day or not), sleep time (<6 hours/day or not), perceived mental stress (yes or no), having a positive or optimistic mindset/view (yes or no), and history of diabetes mellitus (yes or no), autoimmune disorders (yes or no), and varicella (yes or no). These were chosen as confounding variables, indicated from previous studies.^4^^–^^9^^,^^14^

The incidence of HZ was described per 1,000 person-years, and Kaplan-Meier survival curves were plotted. The Cox proportional hazard regression model was used to obtain hazard ratios (HRs) and 95% CIs of HZ incidence by BMI category. The reference group was the normal-weight group, and the analysis was adjusted for the same variables as in the cross-sectional analysis.

All statistical analyses were two-tailed, and *P* < 0.05 was considered statistically significant. The data were analyzed using the Statistical Package for the Social Sciences software for Windows v. 9.1 (International Business Machines Corporation, Japan Ltd, Tokyo, Japan).

## RESULTS

Table 1 shows total and sex-specific mean values and proportions of baseline characteristics by BMI category. For total participants, compared with the normal-weight group, the underweight group was older, drank alcohol ≥23 g ethanol per day, exercised ≥3 hours per week, walked ≥1 hour per day, had the lower proportions of people with a history of diabetes, and had a higher proportion of people with a history of autoimmune disease. In contrast, the overweight group had the lower proportions of current smokers and those who exercised ≥3 hours per week, walked ≥1 hour per day, slept for <6 hours a day and had a higher proportion of people with a history of diabetes. The group of overweight men was younger and contained a lower proportion of current smokers, and those who walked ≥1 hour per day, and a higher proportion of those sleeping <6 hours per day, with an optimistic mindset, and a history of diabetes mellitus than normal-weight men. Underweight men were older, and the group contained a lower proportion of those drank alcohol, exercised ≥3 hours per week, walked ≥1 hour per day, and had optimistic views than the group of normal-weight men. The group of overweight women contained a lower proportion who exercised ≥3 hours per week and walked ≥1 hour per day, and a higher proportion of those sleeping <6 hours per day, having optimistic views, and a history of diabetes mellitus than normal-weight women. Underweight women were older, and the group had a higher proportion of current smokers, those sleeping <6 hours per day, and those with a history of autoimmune disorder, and a lower proportion who exercised ≥3 hours per week, walked ≥1 hour per day, and had optimistic views than the group of normal-weight women.

**Table 1.** Total and sex-specific mean values and proportions of baseline characteristics by body mass index category

	Body mass index, kg/m^2^		

	Underweight (<18.5)	Normal weight (18.5 to <25.0)	Overweight (≥25.0)
Total			
Number of participants (*n* = 12,311)	981	8,408	2,922
Body mass index, kg/m^2^, mean (SD)	17.2 (1.1)^c^	21.9 (1.7)	27.3 (2.3)^c^
Age, years, mean (SD)	72.3 (11.6)^c^	67.6 (10.5)	66.4 (9.9)^c^
Current smoker, %	16.8	17.9	16.2^a^
Alcohol intake ≥23 g/day, %	12.6^c^	21.2	22.7
Exercise ≥3 hours/week, %	22.7^c^	29.8	26.1^c^
Walking ≥1 hour/day, %	41.9^c^	49.4	44.5^c^
Sleep time <6 hours, %	13.5	11.7	14.3^c^
Perceived mental stress, %	20.5	18.9	20.0
Optimistic mindset/views, %	41.6^c^	52.1	57.0^c^
History of diabetes mellitus, %	6.8^a^	9.0	13.5^c^
History of autoimmune disorders %	3.7^b^	2.1	1.6
History of varicella, %	14.8	16.2	16.0

Men			
Number of participants (*n* = 5,492)	336	3,732	1,424
Body mass index, kg/m^2^, mean (SD)	17.4 (0.9)^c^	22.1 (1.7)	27.2 (2.2)^c^
Age, years, mean (SD)	72.3 (10.9)^c^	66.9 (10.1)	65.0 (9.4)^c^
Current smoker, %	37.5	36.1	30.2^c^
Alcohol intake >23 g/day, %	31.6^c^	43.3	43.2
Exercise ≥3 hours/week, %	24.7^b^	32.5	29.8
Walking ≥1 hour/day, %	36.6^c^	48.2	43.1^c^
Sleep time <6 hours, %	7.4	9.9	12.3^a^
Perceived mental stress, %	16.7	17.4	18.9
Optimistic mindset/views, %	40.5^c^	52.3	55.4^a^
History of diabetes mellitus, %	8.6	11.2	14.5^c^
History of autoimmune disorders, %	1.8	1.1	1.1
History of varicella, %	12.8	11.7	12.6

Women			
Number of participants (*n* = 6,819)	645	4,676	1,498
Body mass index, kg/m^2^, mean (SD)	17.1 (1.1)^c^	21.8 (1.7)	27.4 (2.3)^c^
Age, years, mean (SD)	72.3 (12.0)^c^	68.1 (10.8)	67.8 (10.2)
Current smoker, %	6.0^b^	3.5	3.0
Alcohol intake >23 g/day, %	2.8	3.6	3.1
Exercise ≥3 hours/week, %	21.6^b^	27.6	22.5^c^
Walking ≥1 hour/day, %	44.7^b^	50.2	45.8^b^
Sleep time <6 hours, %	16.6^a^	13.2	16.2^b^
Perceived mental stress, %	22.0	20.0	21.0
Optimistic mindset/views, %	41.9^c^	51.9	58.4^c^
History of diabetes mellitus, %	5.9	7.3	12.6^c^
History of autoimmune disorders, %	4.7^b^	2.8	2.2
History of varicella, %	15.8	19.7	19.2

SD, standard deviation.

Difference from normal-weight group: ^a^*P* < 0.05, ^b^*P* < 0.01, ^c^*P* < 0.001.

Table 2 shows ORs (95% CIs) of past history of HZ by BMI category among all participants, men and women. The ORs of HZ prevalence for underweight and normal weight were statistically insignificant in all participants, men and women using both age-adjusted and multivariate-adjusted models. In all participants, the ORs of HZ prevalence for overweight compared with normal-weight groups were 1.17 (95% CI, 0.79–1.72) in the multivariate model; the corresponding ORs were 1.30 (95% CI, 0.72–2.33) in men and 1.07 (95% CI, 0.63–1.80) in women.

**Table 2.** Odds ratios of past history of herpes zoster by body mass index category in all participants, men and women

	Body mass index (kg/m^2^)		

	Underweight (<18.5)	Normal weight (18.5 to <25.0)	Overweight (≥25.0)
Total participants (*n* = 12,311)	981	8,408	2,922
Number of cases (prevalence, %)	135 (13.8)	1,257 (15.0)	426 (14.6)

Age- and sex-adjusted odds ratio (95% CI)	0.96 (0.48–1.89)	1.00	1.20 (0.82–1.76)
*P*-value	0.90		0.34
Multivariable odds ratio (95% CI)^a^	0.97 (0.49–1.92)	1.00	1.17 (0.79–1.72)
*P*-value	0.93		0.43

Men (*n* = 5,492)	336	3,732	1,424
Number of cases (prevalence, %)	37 (11.0)	398 (10.7)	159 (11.2)

Age-adjusted odds ratio (95% CI)	1.80 (0.71–4.60)	1.00	1.28 (0.72–2.28)
*P*-value	0.22		0.40
Multivariable odds ratio (95% CI)^a^	1.82 (0.70–4.74)	1.00	1.30 (0.72–2.33)
*P*-value	0.22		0.39

Women (*n* = 6,819)	645	4,676	1,498
Number of cases (prevalence, %)	98 (15.2)	859 (18.4)	267 (17.8)

Age-adjusted odds ratio (95% CI)	0.56 (0.20–1.59)	1.00	1.13 (0.67–1.88)
*P*-value	0.28		0.66
Multivariable odds ratio (95% CI)^a^	0.55 (0.19–1.58)	1.00	1.07 (0.63–1.80)
*P*-value	0.27		0.81

CI, confidence interval.

^a^Adjusted for age, smoking, drinking, exercise, walking, sleep time, perceived mental stress, optimistic view, and history of diabetes mellitus, autoimmune disorder, and varicella.

**Figure 2.**
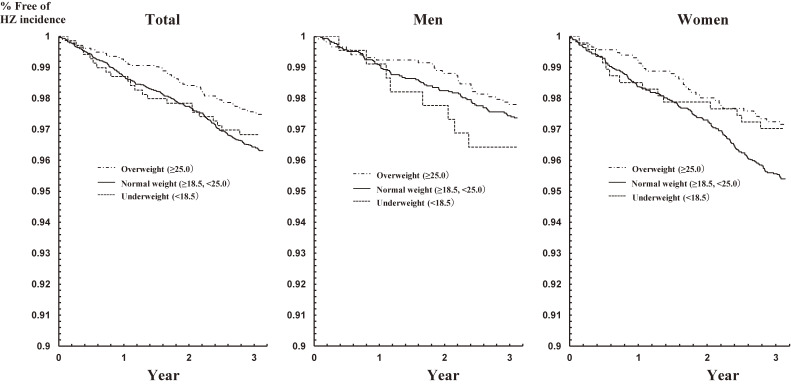
Kaplan-Meier plots of HZ incidence during the 3-year observational period among the three body mass index groups in all participants, men, and women

Figure 2 shows the Kaplan-Meier estimates for HZ incidence during the 3-year observational period by BMI category. For all participants, the log-rank test showed a significant difference in the estimates between the normal-weight and overweight groups (*P* < 0.01), but there was no significant difference between the underweight and normal-weight groups.

In men, the result of the log-rank test was insignificant between the normal-weight and overweight groups (*P* = 0.43), although the proportion of HZ incidence in the overweight group tended to be higher than in the normal-weight group (closed line in Figure 2). In women, the result of the log-rank test was significant between the normal-weight and overweight groups (*P* < 0.01), but insignificant between the normal-weight and underweight groups.

Table 3 shows HRs (95% CIs) of HZ incidence by BMI category in all participants, men and women. A test for non-proportionality showed the absence of time dependency, confirming proportionality. The HRs of HZ incidence for the underweight versus normal-weight groups were marginally significant in women, but not for all participants or men. The multivariate HRs for the underweight and overweight groups versus normal-weight group in women were 0.58 (95% CI, 0.34–1.01) and 0.57 (95% CI, 0.39–0.83), respectively.

**Table 3.** Hazard ratios of incident herpes zoster by body mass index category in all participants, men and women

	Body mass index (kg/m^2^)		

	Underweight (<18.5)	Normal weight (18.5 to <25.0)	Overweight (≥25.0)
Total participants			
Person-years	2,368	21,178	7,482
Number of cases	22	258	61
Incidence, per 1,000 person-years	9.3	12.2	8.2

Age- and sex-adjusted hazard ratio (95% CI)	0.71 (0.45–1.09)	1.00	0.69 (0.52–0.92)
*P*-value	0.12		0.01
Multivariable hazard ratio (95% CI)^a^	0.72 (0.46–1.12)	1.00	0.67 (0.51–0.90)
*P*-value	0.14		<0.01

Men			
Person-years	813	9,874	3,788
Number of cases	8	84	28
Incidence, per 1,000 person-years	9.8	8.5	7.4

Age-adjusted hazard ratio (95% CI)	1.09 (0.52–2.27)	1.00	0.86 (0.56–1.35)
*P*-value	0.82		0.52
Multivariable hazard ratio (95% CI)^a^	1.19 (0.57–2.49)	1.00	0.86 (0.55–1.34)
*P*-value	0.65		0.50

Women			
Person-years	1,555	11,304	3,694
Number of cases	14	174	33
Incidence, per 1,000 person-years	9.0	15.4	8.9

Age-adjusted hazard ratio (95% CI)	0.58 (0.34–1.01)	1.00	0.60 (0.41–0.87)
*P*-value	0.052		<0.01
Multivariable hazard ratio (95% CI)^a^	0.58 (0.34–1.01)	1.00	0.57 (0.39–0.83)
*P*-value	0.06		<0.01

CI, confidence interval.

^a^Adjusted for age, smoking, drinking, exercise, walking, sleep time, perceived mental stress, optimistic view, and history of diabetes mellitus, autoimmune disorder, and varicella.

## DISCUSSION

In a large population-based study among over 10,000 residents aged ≥50 years, we found no association between BMI and past history of HZ in cross-sectional analysis. In the cohort analysis, we found that the overweight groups for all participants and women had a lower incidence of HZ than the normal-weight groups, even after adjustment for potential confounding variables. Men showed a similar association, but it was not statistically significant, partly because the smaller number of HZ cases in men that the test had lower statistical power. Underweight was associated with a lower incidence of HZ among women but not men. However, the multivariate HR of HZ for underweight versus normal-weight decreased with marginal significance, in part due to the small number of cases in the underweight group.

No previous studies have examined the association between BMI and the prevalence or incidence of HZ. Forbes et al investigated the risk factors for HZ prevalence through a case-control study of British adults aged ≥18 years (144,959 cases and 549,336 controls) using a primary case database, but did not describe an association between BMI and HZ.^9^

The lower risk of HZ associated with being overweight can be explained in part by a previous study, which found that enhanced cell-mediated immunology, expressed as higher CD4+ count and CD4+/CD8+ ratio, was positively correlated with BMI.^13^

The lack of association between BMI and past history of HZ in our cross-sectional analysis could be because of the use of BMI at the baseline, not at the time that symptoms of HZ occurred. BMI could change after a previous episode of HZ, so that this non-differential misclassification may lead to dilution of the real association.

This study has three main strengths. First, it had a response rate of nearly 100% (98.3%), assuring unbiased results of community-dwelling individuals. Second, a prospective cohort study with a large sample size of more than 10,000 enabled us to carry out a robust investigation of the possible association between being overweight and the risk of HZ. Third, diagnosis of HZ was accurately conducted by three dermatologists using both clinical signs and symptoms, and varicella-zoster virus identification from venous blood samples.

However, the present study also has some limitations. First, the participants’ height and weight were collected using a self-report questionnaire and were not actually measured. However, BMI estimated using self-reported height and weight has been validated in a previous study of 1,823 Japanese aged 40–68 years, where the self-reported height, weight and BMI were highly correlated with measured values (*r* = 0.96 for height and weight, *r* = 0.92 for BMI). Differences in mean height, weight, and BMI for the actual versus self-reported measures were 0.2 to 0.3 cm, −0.4 to −0.1 kg, and −0.1 to −0.2 kg/m^2^.^17^ Second, the association between being extremely overweight (BMI ≥30 kg/m^2^), and the risk of HZ could not be examined in this study because there were just 272 participants and two HZ cases among people with BMI ≥30 kg/m^2^.

In Japan, the package insert for varicella vaccine was revised in 2004 to include elderly people with reduced immunity to varicella virus, and in March 2016, people over 50 years of age was added as vaccine targets. Our study suggests that overweight is unlikely raise risk of HZ, and thus target for vaccination can be set independent of BMI.

### Conclusion

The SHEZ Study showed that being overweight was associated with a lower incidence of HZ among older adults in the general Japanese population than being normal weight. The association was more evident in women than men. Further studies will be required to determine the effects of being overweight on the reduction of risk of HZ.

## SUPPLEMENTARY MATERIAL

eTable 1. STROBE checklist of items that should be included in reports of cohort studies
